# The Serum Treatment of Cerebro-Spinal Fever

**Published:** 1907-05-18

**Authors:** 


### THERAPEUTICS.

### THE SERUM TREATMENT OF CEREBRO-SPINAL FEVER.

Following the publication last year by Professor Ruppel of his researches upon the meningococcus (diplococcus of Weichselbaum) and his experimental work on animals on the subject of immunisation, a serum has at length been prepared which gives promise of proving effective in the human subject. Ruppel's careful work leaves no doubt that by the subcutaneous injection of his serum into animals (particularly rabbits and porpoises) the animals are rendered immune to subsequent inoculation with the meningococcus or recover from the effects of its previous inoculation; so that in animals, at least, the serum appears to act as reliably as the diphtheria antitoxin.

The potency of the serum appears very great; 0.01 c.c. proved protective against 100 to 1,000 times the fatal dose of meningococcus in porpoises, and 2.5 c.c. counteracted the effect if injected after these inoculations of the culture had been made.

Very few data exist as to the practical results in man, for cerebro-spinal fever fortunately is not always at hand; but from trial in a few instances Professor Ruppel suggests that 25 c.c. will suffice for the curative dose and 5 c.c. for the protective dose.

Subcutaneous injection is the mode of application. One is specially warned that intra-spinal or cerebral injection is most dangerous. When injected under the skin no ill effects are observed.

Meister, Lucius and Brünning have arranged to supply the serum in a desiccated preparation (it is better kept thus) in phials containing 2.5 mg. and 0.5 mg. respectively—the curative and protective dose. For use 25 c.c. of *cold, sterilised* water are added to the phial containing the larger dose, and 5 c.c. of *cold, sterilised* water to the smaller. The phials are constructed to take these quantities, and so allow of the mixing without fear of touching any unsterilised vessels. The injection should be made precisely as in the case of anti-diphtheritic serum.

Although the method is a new and tentative suggestion, yet the disease is so fatal in its results, that no undue rashness is involved in giving the serum a trial during our present widespread epidemic.

